# Incidence of hemi-diaphragmatic paresis with different volumes of local anaesthetics in interscalene brachial plexus block

**DOI:** 10.1186/s44158-026-00351-5

**Published:** 2026-02-07

**Authors:** Rajagopalan Venkatraman, Ravi Saravanan, Annushha Gayathri

**Affiliations:** Department of Anaesthesiology, SRM Medical College Hospital and Research Centre, Faculty of Medicine and Health sciences, SRM Institute of Science and Technology, Kattankulathur, Chengelpattu, Tamil Nadu India

**Keywords:** Diaphragmatic palsy, Interscalene brachial plexus block, Ultrasonography

## Abstract

**Background and aims:**

The incidence of diaphragmatic-palsy following interscalene brachial plexus block (IBPB) is almost 100% where the drug volume plays a significant role. We compared the incidence of hemidiaphragmatic paresis and the success rate following IBPB using three different volumes of local anaesthetics.

**Methods:**

Ninety patients undergoing shoulder and arm surgeries under ultrasound-guided IBPB were randomly allocated into three groups: Group A (10 ml), Group B (15 ml), and Group C (20 ml). The drug administered was 0.75% ropivacaine with 50 mcg dexmedetomidine. The diaphragm excursion was measured before and 30 min after the block on the side of surgery. The incidence of diaphragmatic palsy and its severity were noted. The success rate following block, the onset of sensory blockade, duration of postoperative analgesia, and adverse effects were observed in all three groups. The statistical analysis was done using SPSS software.

**Results:**

The demographic data, duration of surgery, and success rate following block were statistically insignificant. The hemidiaphragmatic paresis (< 25%, 25–75%, > 75%) in Group A (29,1,0), Group B (17,13,0), and Group C (15,8,7) was statistically significant (*P *value < 0.001). The onset of sensory blockade was Group A (7.06 ± 0.73 min), Group B (6.23 ± 0.72 min), and Group C (4.61 ± 0.63 min) with a *P *value < 0.001. The duration of postoperative analgesia in Group A (440 ± 48.42 min), Group B (429 ± 44.48 min), and Group C (411 ± 51.37 min) was statistically insignificant (*P *value-0.072). Five patients in Group C developed hoarseness of voice postoperatively, which was managed conservatively.

**Conclusion:**

Low volume ultrasound guided interscalene block (10 ml) is associated with a lower incidence of hemidiaphragmatic paresis with a similar success rate and duration of postoperative analgesia. Higher volume of the drug yields a faster onset of the sensory blockade.

**Supplementary Information:**

The online version contains supplementary material available at 10.1186/s44158-026-00351-5.

## Introduction

Interscalene brachial plexus block (IBPB) is a reliable and frequently performed nerve block for upper limb surgeries. IBPB is particularly useful for upper arm, shoulder, and lateral end of clavicle surgeries [1]. IBPB effectively blocks superior (C5, C6) and middle (C7) trunks with frequent sparing of the inferior (C8, T1) trunk. IBPB is associated with side effects like hoarseness of voice, Horner’s syndrome, superior laryngeal nerve paralysis (40–60%), and hemidiaphragmatic paresis. The advent of ultrasound has given us the advantage of direct visualisation of roots and trunks, enabling us to use a lower volume of local anaesthetic (LA), improving the safety [2]. The phrenic nerve originates from the C4 with contributions from C3 and C5. The phrenic nerve traverses caudally along the superior and lateral border of the anterior scalene muscle to the middle part of the muscle, where it lies between the scalenus anterior and prevertebral fascia. Here, it is separated from the brachial plexus by a thin fascial layer [3]. This proximity of the phrenic nerve to the brachial plexus explains the nearly 100% incidence of phrenic nerve palsy following IBPB [4].

IBPB is frequently administered in conjunction with general anaesthesia in shoulder surgeries for postoperative analgesia [1, 5]. IBPB is also used as a sole anaesthetic technique for proximal upper limb surgeries [6, 7]. Several studies have shown that 20 to 30 ml of LA was commonly used for performing IBPB [8, 9]. Several studies were performed on comparing different volumes of LA in IBPB with varying incidence of HDP across the studies [3–9]. A systematic review on RCTs reporting HDP following interscalene block showed that the fragility of the data is high, requiring further clinical trials with large sample sizes [10]. However, no study has compared the incidence of hemidiaphragmatic paresis as well as the success rate with different volumes of the same concentration of a LA in IBPB when it is used as the sole anaesthetic technique.

We hypothesised that a reduction in the volume of LA in IBPB would reduce the incidence of hemidiaphragmatic paresis with a similar success rate. The primary objective of this study was to compare the incidence of hemidiaphragmatic paresis following IBPB performed using three different volumes of LA. The secondary objectives were to compare the success rate, the onset of sensory blockade, the duration of postoperative analgesia, and any adverse effects like hoarseness of voice and Horner’s syndrome.

## Methods

This study was a prospective, observer-blinded, randomised controlled study conducted between December 2020 and August 2021 in a tertiary care medical college hospital after Institutional ethical committee approval (SRM medical college hospital and research centre IEC no. 2066/IEC/2020 dt.25/09/2020) and Clinical Trials Registry-India registration (CTRI/2020/12/029528 dt. 03/12/2020). Written informed consent was obtained from all the patients, and the study was conducted following the guidelines of the declaration of Helsinki. Patients in the age group of 18 to 60, American Society of Anesthesiologists’ (ASA) 1 and 2, and weighing between 50 and 100 kg scheduled for humerus fracture fixation surgeries under IBPB were selected for the study. Patients with pre-existing lung diseases or diaphragmatic dysfunction, bleeding disorders, pregnancy, and uncooperative patients were excluded from the study. Ninety selected patients were randomly allocated to three groups by computer-generated random numbers and sealed, opaque envelope method. At the start of the case, the primary investigator opened the envelope sequentially and prepared the drug solution according to the group involved. All the blocks were performed by a single experienced anaesthesiologist who was not involved in the further part of the study. The patients were monitored intraoperatively and postoperatively by another anaesthesiologist who was blinded to the group allocation. The master allocation sequence and block numbers were known only to the primary investigator and concealed from other investigators till the last case was completed.

All the blocks were performed under ultrasound guidance with ropivacaine 0.75% as the LA. Dexmedetomidine 50 mcg (0.5 ml) was used as an adjuvant in all the groups. The total volume administered was 10 ml in group A, 15 ml in group B, and 20 ml in group C. Patients were premedicated with alprazolam 0.5 mg orally 2 h before surgery. The patients were again explained about the procedure, and the intraoperative monitors used were pulse oximetry, electrocardiogram, and non-invasive blood pressure. The patients were placed in the supine position with the head turned to the opposite side and a slight elevation given to the head of the bed. An Ultrasonogram (USG) Machine (Logiq V2, GE Medical Systems, Jiangsu, China), with a 5–13 MHz linear probe was used. A 21 gauge, 50 mm SonoPlex needle was used to administer the block. Under strict aseptic precautions, ultrasound scanning was done to locate the anterior and middle scalene muscles with the brachial plexus between them. After getting good visualisation of the C5, C6, and C7 nerve roots in the interscalene groove, the prefixed volume of LA was administered according to the group involved. All the patients were sedated with midazolam in titrated doses.

Before and 30 min after the block, an independent observer evaluated ipsilateral hemidiaphragmatic movement on deep inspiration by ultrasonography using a 2–5 MHz broadband curved array transducer. Patients were examined in the semirecumbent position and scanned from a subcostal approach using the liver or spleen as an acoustic window. A < 25% reduction in Diaphragmatic Excursion (DE) was taken as mild paresis. Partial diaphragmatic palsy is diagnosed by a reduction in DE of 25–75% and complete palsy is confirmed by a more than 75% reduction in DE or paradoxical cephalad movement of the diaphragm [11].

The sensory blockade was assessed every five min for up to 30 min by the pinprick method in the C5-6 dermatome on a 3-point verbal rating scale: 0—normal sensation, 1—dull sensation (analgesia), and 2—no sensation (anaesthesia). The onset time for a sensory block was defined as the time elapsed between the time of drug administration and the moment when the pinprick test yielded a score of two. Motor blockade was assessed every five min for up to 30 min for shoulder abduction (C5) and forearm flexion (C6) by modified Bromage scale (4—full power, 3—reduced power but able to lift the arm against resistance, 2—able to move the muscle group against gravity but unable to lift the arm against resistance, 1—perceptible muscle contraction, but unable to move on purpose, and 0—unable to move the relevant muscle group). The onset time for motor block was defined as the time elapsed between the time of drug administration and the moment when the modified Bromage score yielded a score of less than two. Failure to reach a sensory score of two and modified Bromage score of less than two within 30 min of the interscalene block was considered to be a block failure. If the block would have failed, general anaesthesia would have been administered to these patients. But results from these patients also would have been included in the final analysis based on intention-to-treat rather than per-protocol basis [12, 13]. The total cases of block failure were noted, and the success rate for different groups was compared. The total duration of surgery was documented.

At the end of the surgery, the patient was shifted to the postoperative ward. The Visual Analogue Scale (VAS) was used to assess pain, and the patient was explained about the score. The patient was administered paracetamol 1 g intravenously (IV) when VAS ≥ 4 and repeated after six hours only when VAS ≥ 4. If the patient has pain even after paracetamol administration, tramadol 100 mg IV was used as a rescue analgesic. The duration of postoperative analgesia was defined as the time taken from the administration of the block to the time of the first request for postoperative analgesia (VAS ≥ 4) [14]. The patients were observed for any other side effects like hoarseness of voice and Horner’s syndrome. The patients were followed up for 24 h.

### Statistical analysis

Data were entered in MS excel spreadsheet 2020 and analysed using Statistical Package for Social Sciences (SPSS) version 22.0 (trial version). Descriptive statistics including proportions, measures of central tendency, and measures of dispersion were used to describe the data. The skewness was checked to determine the normality of distribution. Chi-square and ANOVA test were used to analyse the data. A *P *value of less than 0.05 was considered significant, and *P* value less than 0.001 was considered highly significant.

*Sample size calculation*—Sample size was calculated based on our pilot study on the incidence of hemidiaphragmatic paresis in 30 patients, with 10 in each group (allocation ratio 1:1:1) and the reported incidence was 10% in group A (10 ml), 30% in group B (15 ml), and 60% in group C (20 ml). For the study to have 80% power, alpha error at 0.05 and with effect size of 1.095 based on proportion of variance of incidence—partial ɳ^2^ (eta-square) of 0.5412 using *f*-tests:ANCOVA:Fixed effects, main effects and interactions in G-power software, the sample size was calculated to be 22 patients in each group. To minimise the effect of data loss, we decided to include 30 patients in each group.

## Results

**Fig. 1:**
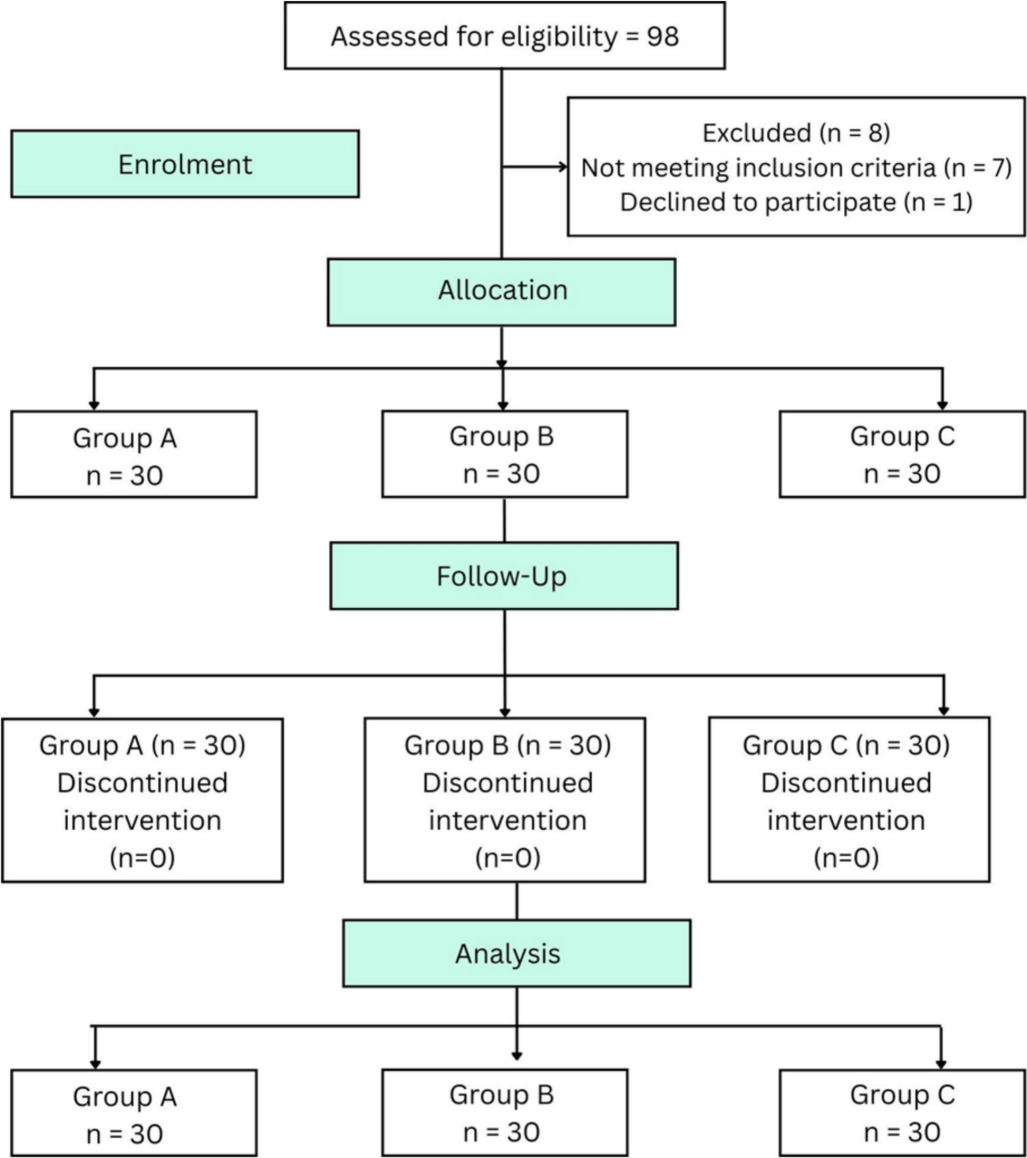
CONsolidated Standards of Reporting Trials (CONSORT) flow chart

**Fig. 2:**
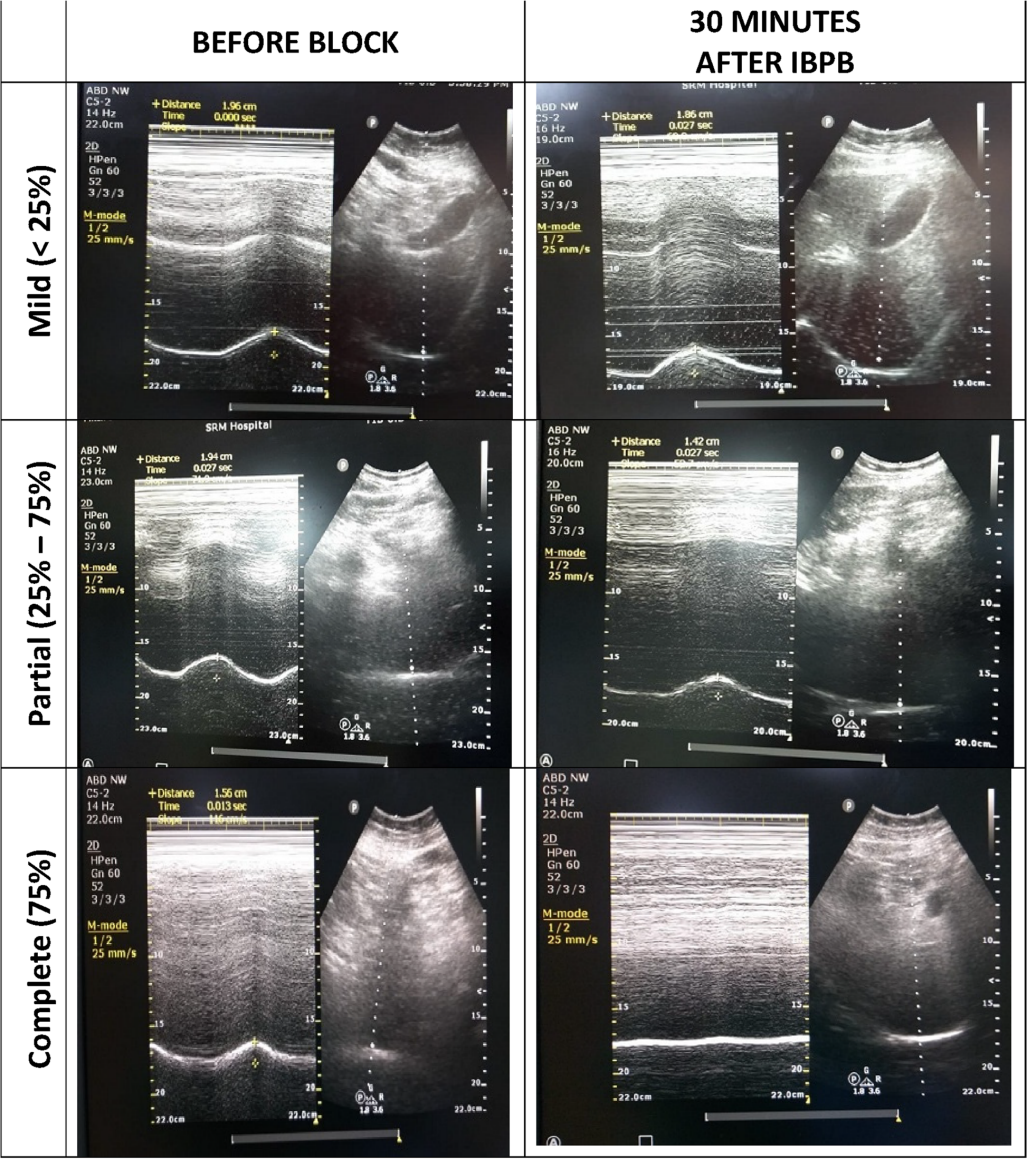
Ultrasound image before and after the IBPB and different degrees of diaphragmatic paresis

Ninety patients enrolled in the study were allocated equally to three different groups. The flow of patients in this study is shown in the CONSORT diagram (Fig. 1). There was no difference in demographic profile, like age, sex, ASA physical status, body weight, as well as the duration of surgery between the groups (Table 1). The baseline diaphragmatic movements were normal in all the patients. In group A, 29 (96.7%) had mild paresis, while one (3.3%) patient had partial diaphragmatic palsy. In group B, 17 (56.7%) patients had mild paresis, and 13 (43.3%) patients developed partial diaphragmatic palsy. Complete palsy was not encountered in any of the patients in either group A or group B. However, 15 (50%) patients developed mild paresis, 8 (26.7%) patients had partial diaphragmatic palsy, and complete palsy was observed in seven (23.3%) patients. Only one patient with complete palsy developed respiratory difficulty with a drop in saturation to 92%. The patient was managed conservatively with reassurance and oxygen through a face mask. The rest of the patients with diaphragmatic palsy did not develop any clinical symptoms. There was a statistical significance in the incidence of hemidiaphragmatic paresis between the three groups (*P* value < 0.001). The ultrasound images of different types of hemidiaphragmatic paresis are shown in Fig. 2.

**Table 1:** Demographic profile

Parameters	Group A	Group B	Group C
Age (years)	37.5 ± 13.14	38.8 ± 12.71	39.16 ± 12.42
Gender (male/female)	16 (53.33%)/14 (46.67%)	17 (56.67%)/13 (43.33%)	13 (43.33%)/17 (56.67%)
Weight (kg)	61.4 ± 5.70	62.6 ± 4.95	62.96 ± 5.18
ASA status (I/II)	6 (20%)/24 (80%)	9 (30%)/21 (70%)	5 (16.67%)/25 (83.33%)
Duration of surgery (min)	143 ± 20.37	141.33 ± 35.21	145.66 ± 37.20

Values are Mean ± SD or number of patients

The success rate was 100% in all three groups. The onset of sensory blockade was shorter in group C (4.61 ± 0.63 min) than in group B (6.23 ± 0.72 min) and group A (7.06 ± 0.73 min). This was statistically significant with a *P* value of < 0.001. The onset of motor blockade was shorter in group C (12.45 ± 0.55 min) than in group B (15.56 ± 0.96 min) and group A (18.15 ± 0.55 min). This was statistically significant with a *P* value of < 0.001. The duration of postoperative analgesia was also longer in group C (440 ± 48.42 min) than in group B (429.33 ± 44.48 min) and group A (411.33 ± 51.37 min). However, there was no statistically significant difference in the consumption of paracetamol for 24 h between the groups. Tramadol was not required in any of the patients. The results are summarised in Table 2. Five patients in group C developed hoarseness of voice and were managed conservatively. No other complications were encountered.

**Table 2:** Anaesthetic data

Parameters	Group A (10 ml)	Group B (15 ml)	Group C (20 ml)	*P* value
Hemi diaphragmatic paresis (< 25%/25–75%/> 75%)	29/1/0	17/13/0	15/8/7	< 0.001*
Success rate	100%	100%	100%	1
Onset of sensory block (minutes)	7.06 ± 0.73	6.23 ± 0.72	4.61 ± 0.63	< 0.001*
Onset of motor block (minutes)	18.15 ± 1.12	15.56 ± 0.96	12.45 ± 0.55	< 0.001*
Duration of postoperative analgesia (minutes)	411.33 ± 51.37	429.33 ± 44.48	440 ± 48.42	0.072
Number of doses of paracetamol (0/1/2)	3/7/20	5/8/17	5/12/13	0.455

Values are Mean ± SD or number or percentage of patients

^*^P value significant

## Discussion

In this prospective, randomised, observer-blinded trial comparing three different volumes of LA on the incidence of hemidiaphragmatic paresis following IBPB, we found that the incidence of hemidiaphragmatic paresis reduces with a reduction in the volume of LA. The success rate was similar between the three groups. The onset of sensory blockade was shorter, and the duration of postoperative analgesia was prolonged with increasing volumes of LA.

There are various modalities available for the assessment of diaphragm palsy. Ultrasound examination of the diaphragm to measure DE has been shown to have high sensitivity (93%) and specificity (100%) in diagnosing phrenic nerve dysfunction [15]. About 96% of patients in group A developed only mild palsy of < 25%. Seven patients in group C developed complete diaphragmatic palsy. Patients with normal pulmonary function are usually asymptomatic, and reassurance and monitoring are usually sufficient. However, this may be detrimental in patients with poor pulmonary function tests. Urmey et al. in their study on healthy volunteers reported a 100% incidence of hemidiaphragmatic paresis following IBPB, describing it as an inevitable consequence [4]. Casati et al. studied the effects of respiratory function after IBPB with 0.5%, 0.75% ropivacaine, and 2% mepivacaine. They reported ipsilateral hemidiaphragmatic paresis, large mean decreases in forced vital capacity (FVC) and forced expiratory volume (FEV1) at 1 s in all the patients [16]. Hemi diaphragmatic paresis reduces FEV1 by 17–37%, FVC by 21–34% and peak expiratory flow rate (PEFR) by 15.4% [17].

Zhai et al. studied the effects of three different volumes and concentrations of ropivacaine in IBPB. They observed that the paradoxical diaphragmatic movements increased from 58% with 0.75% ropivacaine (6.7 ml) to 69% with 0.5% ropivacaine (10 ml) and 70% with 0.25% ropivacaine (20 ml). This study also shows that the incidence of hemidiaphragmatic paresis increases with an increase in the volume of LA [1]. They also found that the onset of motor and sensory blockade was shorter with low volume and higher concentrations of ropivacaine. But in our study, we used the same concentration of ropivacaine in all the groups. The onset of sensory blockade was shorter with a larger volume of LA. They also showed that the pain scores were similar in all the groups. But, in this study, the duration of postoperative analgesia was longer with 20 ml than with 15 and 10 ml of ropivacaine. The postoperative paracetamol consumption was also less in group C than in groups A and B. Zhai et al. reported one case of Horner’s syndrome (1.01%), while we encountered five cases (5.55%), which are managed conservatively [1].

Riazi et al. also reported a 100% incidence of hemidiaphragmatic paresis with 20 ml of 0.5% ropivacaine compared to 45% with 5 ml. They reported a significant reduction in lung volumes, including FEV1, FVC, and PEFR. The pain scores and the total postoperative morphine consumption were similar in both groups. One patient in the 20 ml group developed a fall in oxygen saturation requiring high flow oxygen therapy [3]. Kim et al. investigated the effects of IBPB on hemidiaphragmatic paresis by comparing 10 ml and 15 ml of 0.5% ropivacaine. They observed that the incidence of hemidiaphragmatic paresis was 92.3% with 15 ml and 53.8% with 10 ml. Two patients in the 15 ml group and one patient in the 10 ml group developed dyspnoea, with the 10 ml group patient also having desaturation requiring oxygen therapy [5].

Lim et al. studied the respiratory effects of IBPB and compared it with a suprascapular nerve block (SSB). They concluded that IBPB results in a reduction in FVC by 31.2% and a reduction in diaphragmatic excursion by 85%.with preserved lung functions in SSB [18]. Palhais et al. tested IBPB administration lateral to the brachial plexus sheath and the incidence of hemidiaphragmatic paresis. They reported that the incidence of hemidiaphragmatic paresis was 90% in the conventional technique and 21% in the extrafascial technique. The respiratory functions were well preserved with similar pain scores in the extrafascial technique [19].

Several studies have shown that the incidence of diaphragmatic palsy is related to the volume of LA administered in IBPB. Some studies were done trying to reduce the incidence of hemidiaphragmatic paresis by reducing the volume as well as concentration of LA, trying extrafascial injection of IBPB, and using suprascapular nerve block as an alternative to IBPB [20, 21]. But most of these studies used IBPB only for postoperative analgesia rather than as a sole anaesthetic technique for surgical anaesthesia. The anaesthesiologists are worried about the failure of the block when IBPB is used as a sole anaesthetic technique for surgery. Hence, many prefer to use 15 to 20 ml of LA, which increases the chance of diaphragmatic palsy.

We were able to achieve a 100% success rate even when 10 ml of LA was used. All the blocks in this present study were done by a single experienced anaesthesiologist under ultrasound guidance. This study reiterates that after adequate training in IBPB, a low volume (10 ml) of LA will suffice for the successful blockade.

Our study has a few limitations. The blocks were performed by an experienced anaesthesiologist, and the results may not be reproducible when done by a training doctor. Secondly, we did not do spirometric measurements of pulmonary function. This would have given us the exact restriction of pulmonary functions following hemidiaphragmatic paresis. Finally, although the patient and the assessor were blinded to the study, the anaesthesiologist performing the block was not blinded to the volume of LA administered. The opportunity of reducing the volume of LA to less than 10 ml in IBPB gives us scope for future research in this area.

## Conclusion

The incidence and severity of hemidiaphragmatic paresis reduced with the volume of local anaesthetics in interscalene block, with 10 ml producing only mild paresis and hence may be considered in patients with pulmonary pathology. The success rate was 100% in all the three groups. However, the onset of sensory and motor blockade was shorter, and the duration of postoperative analgesia was prolonged in the 20 ml group.

## Supplementary Information

Additional file 1.

Additional file 2.

## Data Availability

The data used to support the findings of this study were published in Mendeley and are available from the corresponding author upon request.
