# Supplementary material for: Incidence of hemi-diaphragmatic paresis with different volumes of local anaesthetics in interscalene brachial plexus block
Source: J Anesth Analg Crit Care. 2026 Feb 7;6:40. doi: 10.1186/s44158-026-00351-5 (PMC12977868; doi:10.1186/s44158-026-00351-5)
Supplement: Supplementary file 1 — Additional file 1. [file 44158_2026_351_MOESM1_ESM.rtf]

[6] -- Monday, December 29, 2025 -- 14:33:05
F tests - ANCOVA: Fixed effects, main effects and interactions
Analysis:	A priori: Compute required sample size 
Input:	Effect size f	=	1.09
	á err prob	=	0.05
	Power (1-â err prob)	=	0.80
	Numerator df	=	10
	Number of groups	=	3
	Number of covariates	=	1
Output:	Noncentrality parameter ë	=	17.8215000
	Critical F	=	1.6939605
	Denominator df	=	11
	Total sample size	=	15
	Actual power	=	0.8250551
